# Fabrication of GaN nano-towers based self-powered UV photodetector

**DOI:** 10.1038/s41598-021-90450-w

**Published:** 2021-05-25

**Authors:** Lalit Goswami, Neha Aggarwal, Pargam Vashishtha, Shubhendra Kumar Jain, Shruti Nirantar, Jahangeer Ahmed, M. A. Majeed Khan, Rajeshwari Pandey, Govind Gupta

**Affiliations:** 1 CSIR-National Physical Laboratory, Dr K.S. Krishnan Road, New Delhi, 110012 India; 2 Department of Electronics & Communication Engineering, Delhi Technological University, New Delhi, 110042 India; 3 Functional Materials and Microsystems Research Group and the Micro Nano Research Facility, RMIT University, Melbourne, VIC 3000 Australia; 4 Department of Chemistry, College of Science, King Saud University, Riyadh, 11451 Saudi Arabia; 5 King Abdullah Institute for Nanotechnology, King Saud University, Riyadh, 11451 Saudi Arabia; 6 Present Address: Academy of Scientific & Innovative Research, CSIR-HRDC Campus, Ghaziabad, Uttar Pradesh 201002 India

**Keywords:** Electronic devices, Materials for devices

## Abstract

The fabrication of unique taper-ended GaN-Nanotowers structure based highly efficient ultraviolet photodetector is demonstrated. Hexagonally stacked, single crystalline GaN nanocolumnar structure (nanotowers) grown on AlN buffer layer exhibits higher photocurrent generation due to high quality nanotowers morphology and increased surface/volume ratio which significantly enhances its responsivity upon ultraviolet exposure leading to outstanding performance from the developed detection device. The fabricated detector display low dark current (~ 12 nA), high I_Light_/I_Dark_ ratio (> 10^4^), fast time-correlated transient response (~ 433 µs) upon ultraviolet (325 nm) illumination. A high photoresponsivity of 2.47 A/W is achieved in self-powered mode of operation. The reason behind such high performance could be attributed to built-in electric field developed from a difference in Schottky barrier heights will be discussed in detail. While in photoconductive mode, the responsivity is observed to be 35.4 A/W @ − 3 V along with very high external quantum efficiency (~ 10^4^%), lower noise equivalent power (~ 10^–13^ WHz^−1/2^) and excellent UV–Vis selectivity. Nanotower structure with lower strain and dislocations as well as reduced trap states cumulatively contributed to augmented performance from the device. The utilization of these GaN-Nanotower structures can potentially be useful towards the fabrication of energy-efficient ultraviolet photodetectors.

## Introduction

Gallium nitride (GaN) ultraviolet (UV) photodetectors (PDs) attracted a lot of attention due to their versatility and ability to serve in an extreme environment. Thereby, GaN-material inherits the detector properties such as high carrier mobility, high saturation velocity, good-temperature sustainability, high breakdown voltages, inertness and hardness towards chemical and radiation respectively^1^. With all these advantageous properties, GaN UV-PD have established its position in numerous applications such as monitoring of combustion, flame detection, ozone detection, space communication, biological sensors: phototherapy, sterilization control, solar irradiance measurement, industrial monitoring, missile plume detection and ultraviolet astronomy etc^2^. So far, various UV-PD structures such as p–π–n PDs^3^, p–i–n photodiodes^4^, p–n junction photodiodes^5^, Schottky-barrier photodiodes^6,7^, and metal–semiconductor–metal (MSM) PDs^8,9^, have been fabricated. The ease of fabrication and rapid time-correlated response ascertained the advantage of Schottky barrier type and MSM structured UV-PDs as compared to the p–n junction. Recent reports also suggest that extremely low dark current has been achieved with MSM based UV-PDs^10^. Since the reduction in dark current is affected by low defect density, thus device performance can be directly correlated with defects generated during the growth of device structure. Simultaneously, these defects can induce stress/strain which are accredited to lattice mismatch, and thermal expansion coefficient difference between GaN and the substrate^11^. Further, in the process of PDs improvisation, nanostructures (NS) have played a significant role due to very low stress–strain induced electrical as well as optical defects that reciprocate its significant impact on detector performance^12,13^. Moreover, NSs were also recognized as a structure with high surface to volume ratio i.e., higher aspect ratio which can provide more absorption sites to incident photons for generating high density of charge carriers, and lower resistance path which can cumulatively increase the detection capability of the PDs. Therefore, growth of high surface-to-volume ratio and stress relaxed 3D GaN-NSs could play a crucial role in the development of highly efficient optoelectronic devices. Recently, it has been reported that GaN-nanocolumnar structure offers wavelength tunability, low dislocation density and lower lattice related strain along with very good response^14–16^. However, the fabricated devices are reported to be non-operational in photovoltaic mode i.e. zero applied bias which will be explored and realized in the present study. Along with this, in the non-GaN PDs domains, the present study will be demonstrating better performance in self-powered mode as compared to solar blind ZnO–Ga_2_O_3_ heterostructure and Ru decorated AlGaN nanowire based UV-PD^17,18^.

In this letter, we report the utilization of AlN buffer layer (800 °C) for growth of less strained and stress relaxed, higher aspect ratio hexagonally stacked GaN-nanocolumnar structures i.e. nanotowers (GaN-NTs) on Si (111) substrate via plasma assisted molecular beam epitaxy (PAMBE) system. The impact of buffer layer on the quality of top epitaxial layer in the grown heterostructure is illustrated along with the fabrication of UV photodetector. The performance of the fabricated UV-PD is analysed based on the parameters such as responsivity, detectivity, noise equivalent power (NEP) and external quantum efficiency (QE) under UV illumination. Besides, the spectral selectivity of the fabricated UV-PD for the wavelength ranging from 200 to 800 nm under variable applied bias has also been assessed. Moreover, the presented work can be motivated to host new generation UV-PDs where grown novel GaN-NTs structure can be utilized as a template to fabricate recombination supressed, heterojunction based more efficient^19^ and wearable^20^ UV-PDs.

## Experimental

**Figure 1:**
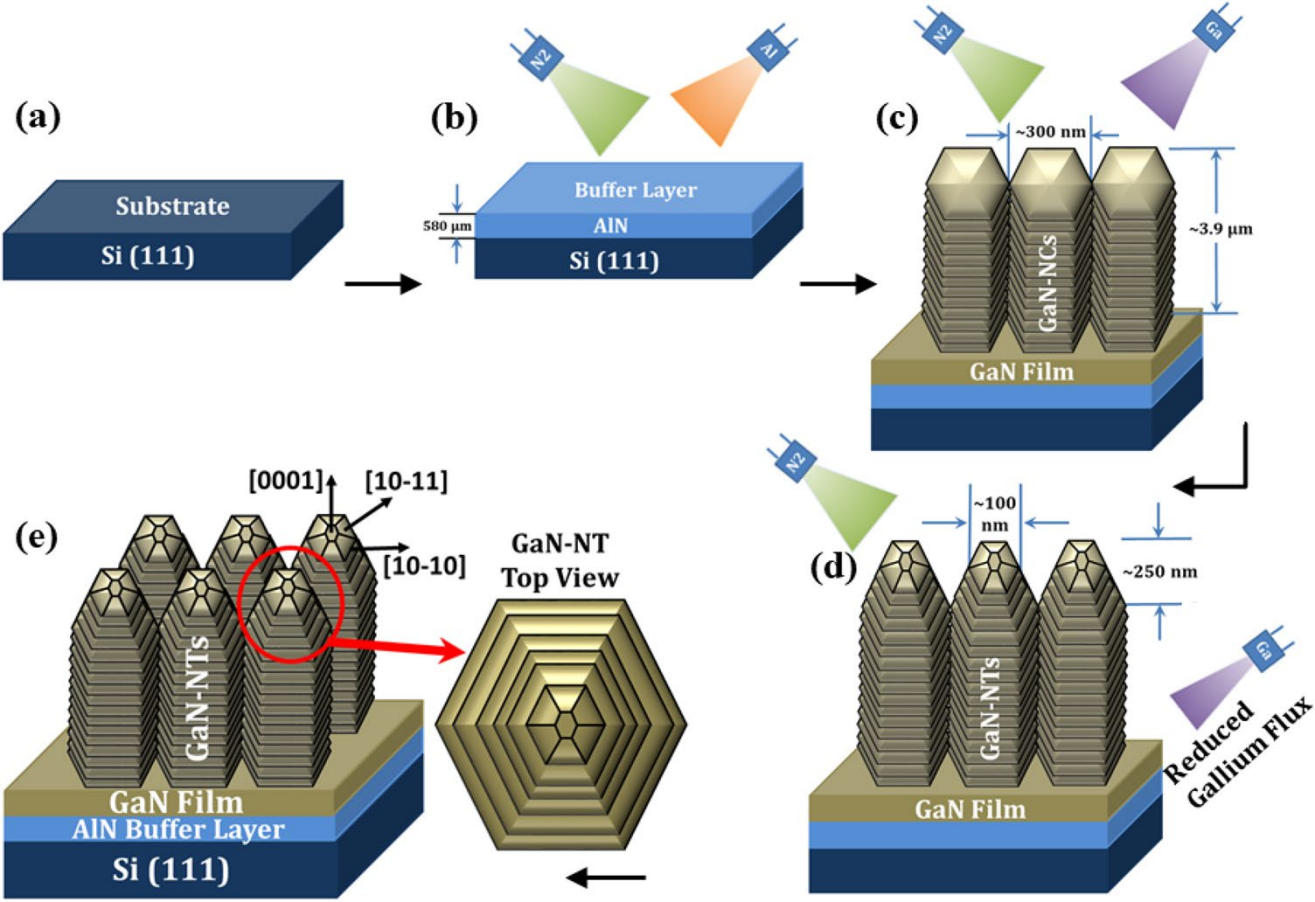
(**a**–**e**) A schematic growth diagram showing the growth of GaN NTs on Si (111) substrate with sandwitched AlN buffer layer in between.

A unique structure (as shown in Fig. 1) i.e. GaN-NT arrays have been grown epitaxially via PAMBE (Compact 21 Riber) system. After standard wet chemical etching and thermal cleaning procedure^21^ of Si (111) substrate (Fig. 1a), the initial growth of AlN buffer layer has been performed at 800 °C for 30 min with Al beam equivalent pressure (BEP) of ~ 6.8 × 10^−7^ Torr and growth rate of 3.2 Å/s (shown in Fig. 1b). Afterwards, a wetting layer of Gallium (Ga) has been deposited for 10 s at a lower substrate temperature of 666 °C that precedes the nucleation process of GaN growth. Through the heat energy gained by providing appropriate substrate temperature, Ga molecules and N_2_ ion active species acquire enough energy at the substrate surface to forms GaN compound. The growth of GaN-NTs has been performed at 666 °C substrate temperature for 210 min. with Ga BEP of 2.8 × 10^−6^ Torr. To fulfil the essential requirement for nanocolumnar GaN growth, nitrogen-rich condition (III/V ratio < 1) was maintained by adjusting N_2_ flow up to 3 sccm with 400 W RF-plasma power with growth rate 3.2 Å/s. Thereafter, the growth conditions of closely packed GaN-NCs (shown in Fig. 1c) has been altered to the taper-ended geometry of nanotower-like structure by gradually reducing the Ga-flux from 2.8 × 10^−6^ to 1.5 × 10^−6^ Torr for next 600 s during the growth process (as shown in Fig. 1d–e).

The real-time monitoring of GaN-NTs/Si (111) growth has been performed by reflection high electron diffraction (RHEED) using in-situ STAIB electron gun (12 keV). The morphological studies of grown GaN-NTs have been carried out by using Field-emission scanning electron microscopy (FEI Verios 460L FEGSEM) and Atomic force microscopy (AFM) (Multimode-V Veeco). The structural quality of the grown structure was examined by High-Resolution X-ray Diffraction (HRXRD) (PANalyticalX’Pert PRO MRD System) using CuKα1 radiation (λ = 1.5406 Å). While, for analysing the optical properties, room temperature (RT) photoluminescence (PL) measurement was carried out by using FLS980 D2D2 (Edinburg) system with 325 nm excitation source (Hd–Cd Kimmon laser) and Raman spectroscopy was carried out in backscattering configuration, using a 514 nm argon-ion laser. The grown GaN-NTs were utilized to fabricate UV-PD in MSM structure by depositing metal electrodes of Au (~ 200 nm)/Cr (~ 10 nm) using thermal evaporation system. Photoresponse measurements were carried out using a probe station setup (S10 Triax probe station with Keithley 2401 acquisition unit with an accuracy of ± 2 nA in current values and ~ 0.05 ms in response time evaluation) on the fabricated device with 1.18 × 10^–3^ cm^2^ active area. The spectrometer is equipped with a focused laser source (λ = 325 nm with the utilized power density of 2.5 mW/cm^2^ to 184 mW/cm^2^).

## Results and discussion

**Figure 2:**
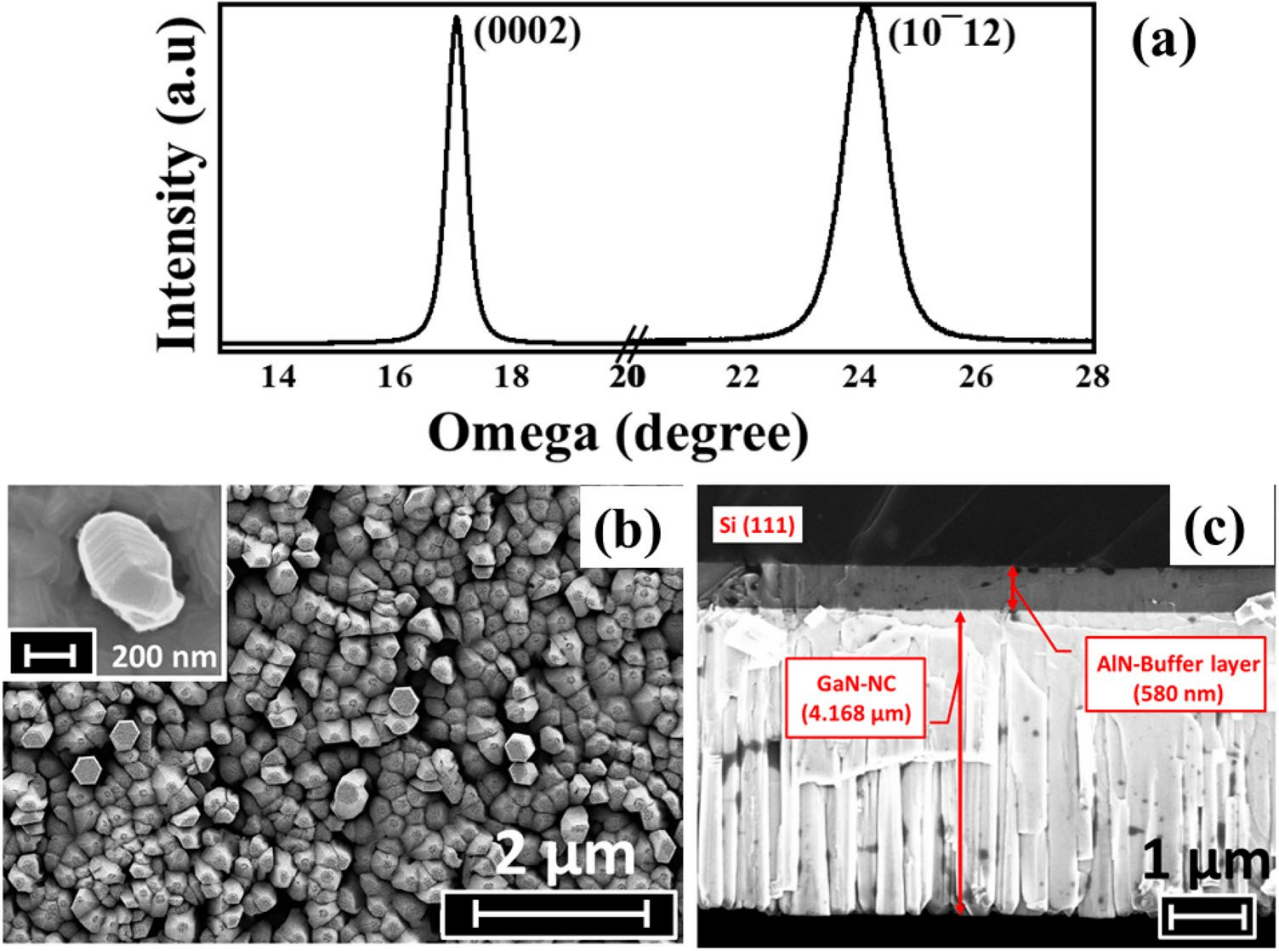
(**a**) HRXRD omega scan along (0002) & (10–12) plane of diffraction, (**b**) Planar view FESEM image (inset: shows a single NT), (**c**) Cross-sectional FESEM image of GaN NTs.

The growth of GaN-NT structure is carried out on reconstructed Si (111) 7 × 7 surface and in-situ monitored by RHEED technique (figure S1, supplementary information).The crystallinity of grown structure was evaluated by HRXRD 2θ–ω scan and its peak positions (figure S2; supplementary information) were utilized to calculate the strain via lattice constant estimation using Bragg’s law^22^. The calculated value of strain in the grown heterostructure is realized to be 0.25% which confirms strain relaxation in the grown NTs. Further, to analyse the dislocation density, HRXRD omega scans along (0002) and (10–12) plane of diffraction have been performed as shown in Fig. 2a. The FWHM values of omega scan along (0002) and (10–12) were found to be 0.39° and 0.84° attributing to screw dislocation density of ~ 1.91 × 10^9^ cm^−2^ and edge dislocation density of ~ 2.33 × 10^10^ cm^−2^_,_ respectively. These dislocation densities are found to be lower in comparison to the previously reported GaN NTs structure grown on Si (111) substrate with thicker AlN buffer layer (665 nm) at a lower growth temperature (762 °C)^16^.

The surface morphology of the grown GaN-NTs has been probed by FESEM (as shown in Fig. 2b) where the planar view of closely packed GaN-NTs with hexagonal shaped nanocolumns was observed with tapered geometry. Inset of Fig. 2b demonstrates a higher magnification image of layer by layer growth of single GaN-NT. Figure 2c displayed the cross-sectional view of the novel hexagonal GaN-NTs having length of ~ 4.168 µm grown along the direction perpendicular to the substrate with AlN-buffer layer (~ 580 nm) sandwiched between them. The notable layered structure is having a stack of uniform hexagonal GaN layers up to the length of ~ 4 µm. Afterwards, lowering of Ga flux resulted in a gradual decrease in the surface area of the stacked top GaN layers which guided towards the formation of unique nanotowers like structure having taper-ended geometry with a tip width of ~ 100 nm. The distribution density of grown NTs is calculated to be 3.5 × 10^10^ cm^−2^. It was also observed that the high quality AlN buffer layer grown at higher substrate temperature (800 °C) has resulted in better structural and morphological properties in comparison to the heterostructure grown with a lower substrate temperature of AlN buffer layer^15,23^. Besides this, the increment in surface area is evaluated in concurrence with FESEM&AFM measurements which confirms the enhancement from 9 to 15.6 µm^2^ from planar to taper-ended GaN-NTs morphology.

**Figure 3:**
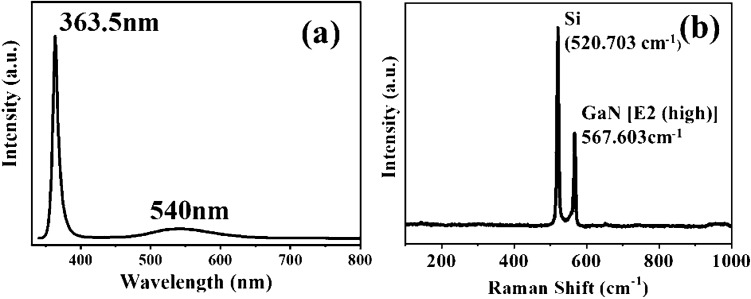
(**a**) RT-PL spectra and (**b**) Raman spectra of as-grown GaN-NTs.

The grown GaN-NTs with enhanced surface area consequently allow more photon absorption sites to generate a large number of photo-excited electron–hole pairs (EHPs). These EHPs emit high-intensity photoluminescence upon recombination which was reflected by intense near band edge emission (NBE) peak at 363.5 nm in RT-PL spectra shown in Fig. 3a. The NBE peak is found to be in close agreement with bulk GaN PL emission (~ 364 nm i.e., 3.406 eV)^24^. The FWHM value of NBE peak is found to be ~ 8.51 nm. The sharp NBE peak with low FWHM value endorse the good crystalline quality of the grown GaN-NTs^25^. An additional peak at ~ 540 nm is also observed in the PL spectra which is attributed to yellow band emission (YBE) originated due to the point defects generated during the growth^26,27^. Furthermore, Raman scattering measurement was employed to quantify the stress in the grown structure as shown in Fig. 3b. The Raman spectra exhibitsE_2_(high) mode of grown GaN-NTs located at 567.603 cm^−1^ which is shifted by ~ 0.397 cm^−1^ as compared to the E_2_ (high) phonon mode of bulk GaN (568 cm^−1^)^24^. The observed red-shift in the E_2_ (high) position signifies the existence of tensile stress in the grown GaN-NTs structure^28,29^. The value of tensile stress^22^ is estimated to be 0.092 GPa which is significantly lower as compared to few previously reported 3D GaN-NSs and 2D GaN films^30–32^ which can be attributed to lower defect states. As, it has been proposed by Hanzaz et al. that these defects can have an adverse impact on electrical as well as the optical properties of the GaN-based UV PDs^33^. Thus, the lower defects in the grown heterostructure attributes to lower trap states, hence it can significantly be employed for fabrication of efficient optoelectronic devices.

**Figure 4:**
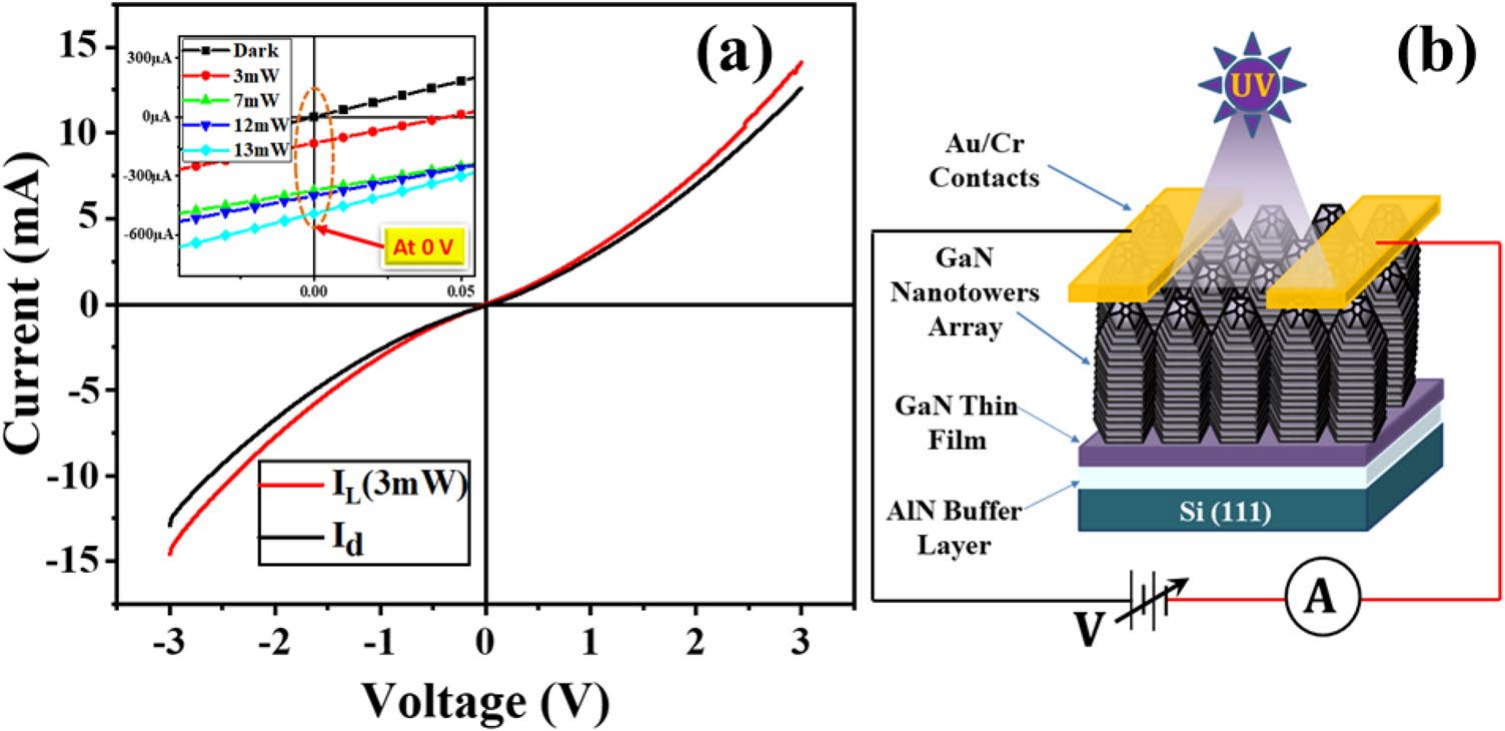
(**a**) RT I-V characteristics of fabricated GaN-NTs based UV-PD under dark and 325 nm UV illumination with 3 mW optical power. Inset: indicate self-powered characteristic at various optical power, (**b**) Schematic image of the fabricated GaN-NT array based device.

Figure 4a displays RT current–voltage (I–V) characteristic of fabricated GaN-NTs based UV-PD under dark as well as 325 nm UV-laser illumination (3 mW optical power) with applied voltage sweep of ± 3 V. The difference in dark and light I–V plots of the fabricated device leads to strong photodetection aspect upon UV illumination. As revealed by examining the I–V plots more closely, the self-channelization of light current (inset Fig. 4a) without applying bias (zero bias) viz. photovoltaic mode, designates the fabricated detector as self-powered UV-PD. The presence of non-identical end tips of the grown GaN-NT structure lead to form non-homogeneous Au/GaN interfaces between Au-metal electrodes and GaN-NTs which exhibits two Schottky natured electrodes with different barrier heights. The variation in barrier heights created a built-in potential gradient and powered the device to respond even without any bias^34^.

**Figure 5:**
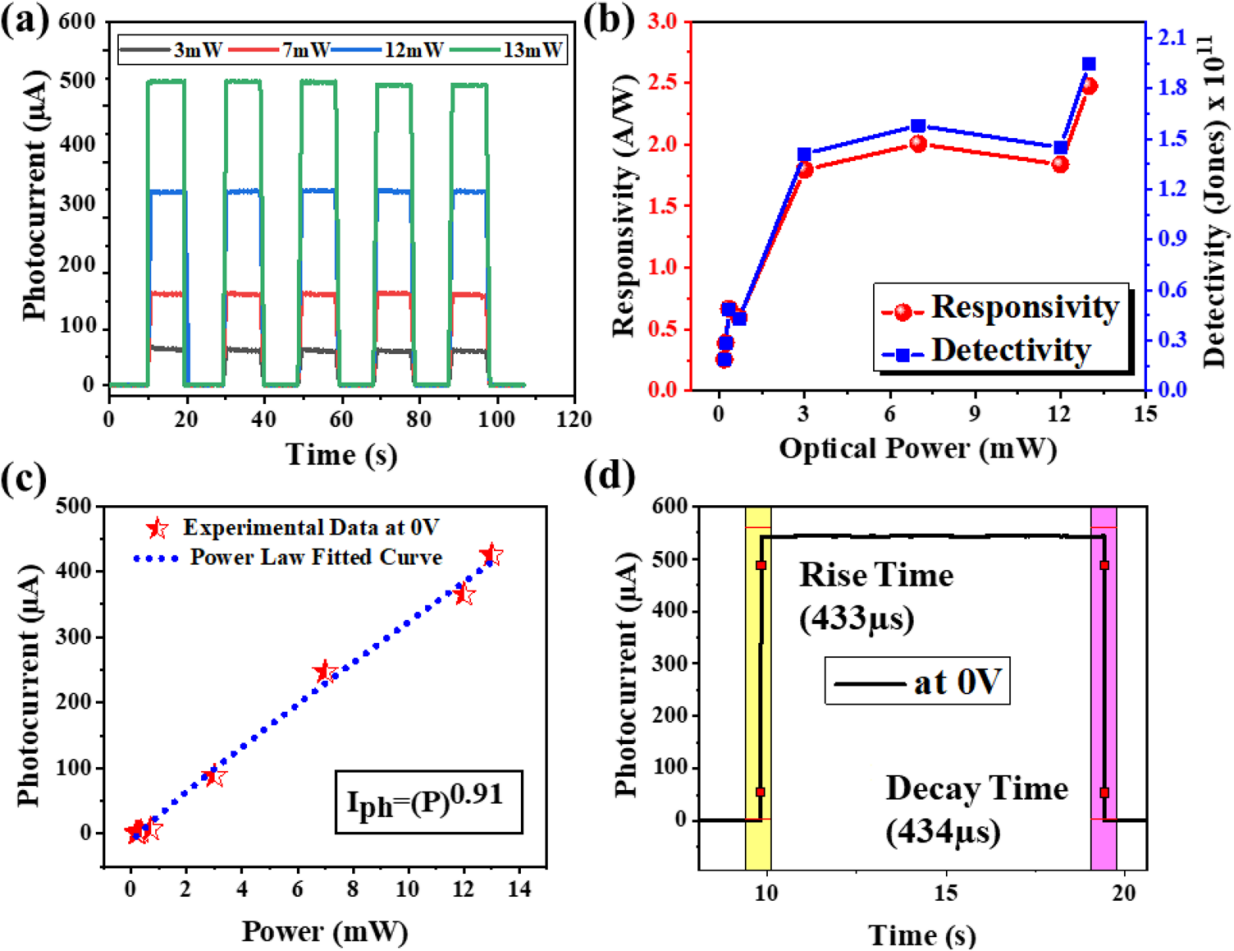
Incident optical power-dependent performance of GaN-NTs UV-PD in photovoltaic mode (**a**) time-correlated transient photoresponse, (**b**) Correlation of responsivity and detectivity with varied optical power, (**c**) Power-law fitted curve in the experimental data of photocurrent, (**d**) Reversibility (response time) of the detector under constant UV illumination with 13 mW optical power.

The schematic image of the fabricated GaN-NT array based device under illumination is shown in the Fig. 4b. Initially, the transient photo-response behaviour of the fabricated detector has been realized in photovoltaic mode with variable incident optical power (from 3 to 13 mW) as shown in Fig. 5a. The transient photo-response measurement has been performed by measuring the inflow current with periodically switching ON (I_Light_) and OFF (I_Dark_) the UV laser source at variable optical power. The maximum value of I_Light_/I_Dark_ ratio was recorded to be > 10^4^ under 13 mW optical power. The analysis demonstrates a very low and consistent dark current of ~ 12 nA, while the fabricated UV-PD shows a stable and significant enhancement in light current value from 67.5 to 545 µA as function of optical power varying from 3 to 13 mW, respectively. Further to explore the capability of the detector to detect low power signals, the optical power is reduced in a range from 0.7 mW to 180 µW and corresponding light current values were observed to be varying from 3.4 µA to 70.8 nA (Figure S3, supplementary information).Conclusively, the fabricated GaN-NTs based UV-PD has shown remarkable (~ 7700 times) enhancement in the photocurrent value from 70.8 nA to 545 µA by varying optical power from 180 µW to 13 mW in the photovoltaic mode of operation.

In addition, the performance of the fabricated device has also been investigated based on their well-known figure-of-merits such as responsivity (R), NEP, detectivity (D), QE (η) and response time by using the following equations^35^:

1 $$R=\frac{{I}_{ph}}{{P}_{inc} \times {A}_{0}}$$

2 $$NEP=\frac{\sqrt[]{2e{I}_{d}}}{R}$$

3 $$D=\frac{R \times \sqrt[]{{A}_{0}}}{NEP}$$

4 $$\eta =\frac{R\times h\times c}{e\times \lambda }$$

where *P*_*inc*_ stands for optical power density of the incident UV photons illuminated on the detector’s active area (*A*_*o*_), *I*_*ph*_ and *I*_*d*_ is the measured photocurrent and dark current, respectively, *h* is the Planck’s constant, *c* is the speed of light, *e* is elementary charge and *λ* is incident UV photon wavelength. The curves in Fig. 5b unveils that the fabricated GaN-based detector yielded a gradual increment in responsivity from 0.255 A/W to 2.47 A/W and detectivity from 1.8 × 10^10^ jones to 1.95 × 10^11^ jones with increasing incident optical power from 180 µW to 13 mW, respectively. Table 1 consolidates the comparison of performance parameters of other GaN based UV-PD operable in self-powered mode of operation. Here, the evaluated responsivity and detectivity is found to be significantly high as compared to the existing bare GaN-based UV-PDs operated in photovoltaic mode of operation which is clearly associated with the stress relaxed growth of unique morphology in the GaN-NT heterostructure. The analysis also divulges a linear relationship between photocurrent and incident optical power as demonstrated in Fig. 5c. The power law has been fitted on the experimental data, *I*_*Ph*_ α *P*^*θ*^, where *θ* determines the photocurrent response w.r.t. optical power density. Here, the *θ* value is determined to be 0.91, which is close to unity, reflecting that the carrier trap states between the conduction band edge and the fermi level are very low^36^. Moreover, to test the reversibility of the detectors, the rise/decay times of transient photoresponse from the fabricated GaN-based UV-PD has been analysed. The sharp rise time (as measured from 10 to 90%) and decay time (from 90 to 10%) under UV on/off condition was observed which were measured to be ~ 433 μs and ~ 434 μs, respectively as shown in Fig. 5d. Table 1 also compares the rapid rise/decay time from the GaN-NTs/AlN/Si heterostructure based UV PD fabricated in the present study with other reported bare GaN-based PDs and the developed UV-PDs are found to exhibit quite fast response towards UV region. Moreover, beyond the GaN based PDs domain, the superiority of the fabricated detector’s quick activation (rise) and retardation (decay) response found to be faster as compared to highly efficient 2D inorganic perovskite Sr_2_Nb_3_O_10_ (SNO) nanosheets^37^ and Ag doped crossed ZnO nano fiber array homojunction based UV-PDs^38^. The GaN NTs with lower AlN buffer layer thickness and low dislocation density along with high surface to volume ratio leads to reduced resistance path for charge transport resulting in efficient photovoltaic operation^39^.

**Table 1:** Comparison of performance parameters of various GaN-based UV-PDs.

Sample	R (mA/W) at 0 V	R (mA/W) at applied bias	Rise/decay time	QE%	References
GaN-NT/Si(111)	2.47 × 10^3^	3.54 × 10^4^, − 3 V	433 µs / 434 µs	1.35 × 10^4^	This work
GaN-NT/Si(111)	–	8.86 × 10^4^, − 3 V	1.25 s/ 1.15 ms	3 × 10^4^	^15^
GaN-NW/n-Si(111)	–	19, 5 V	2.8 s/3.7 s	6.7	^32^
GaN/Si(111)	–	282, 15 V	–	96.71	^30^
GaN-NW/n-Si(100)	–	68, 5 V	2.4 s/3.1 s	24.4	^32^
GaN-MWA/p-Si	131	–	2 ms / 2 ms	49.98	^40^
GaN/Al_2_O_3_	104	–		36	^41^
GaN/Al_2_O_3_	147	–	0.11 ms/0.12 ms	50.7	^42^
GaN p–i–n/pattern Al_2_O_3_	190	210, − 5 V	–	65	^43^
GaN/Al_2_O_3_	300	–	< 0.1 s	–	^44^

**Figure 6:**
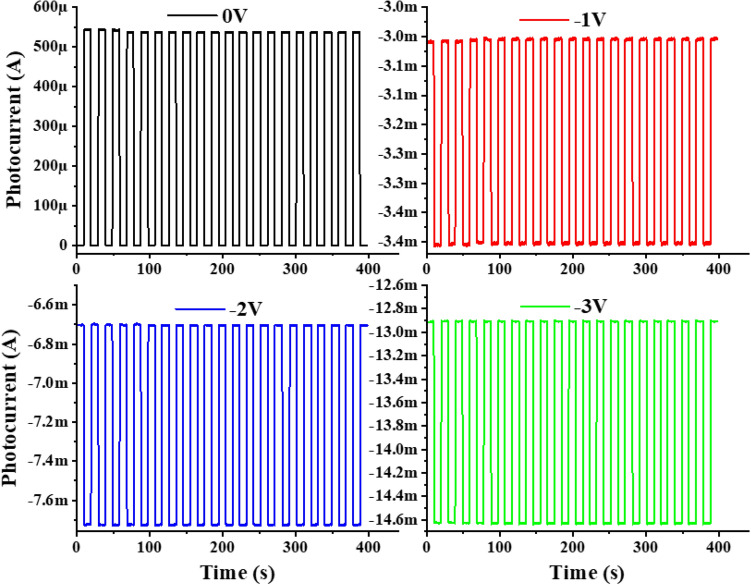
Bias dependent time-correlated transient photoresponse of GaN-NTs UV-PD at 3 mW optical power illumination.

**Figure 7:**
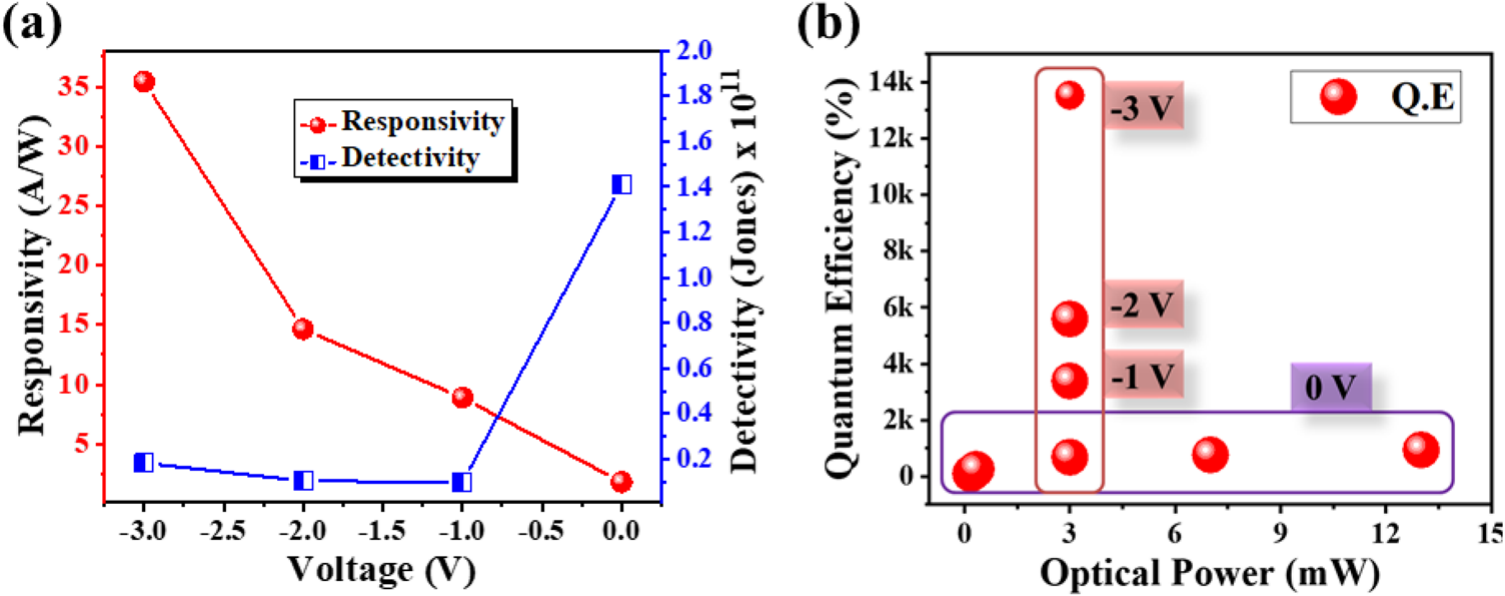
(**a**) Responsivity and detectivity graph w.r.t applied bias, (**b**) QE of fabricated GaN-NTs UV-PD w.r.t incident optical power and applied bias.

To analyse the impact of applied bias on the performance of fabricated GaN-based UV-PD, time-correlated transient response of the detector at a fixed optical power of 3 mW and variable applied bias (from 0 to − 3 V) is acquired as shown in Fig. 6. The investigation revealed an increment in photocurrent values with increasing applied bias i.e., 66.6 µA (0 V), − 0.35 mA (− 1 V), − 1.025 mA (− 2 V) and − 1.73 mA (− 3 V) which is ascribed to enhanced thermionic field emissions. The increment in the photocurrent w.r.t applied bias is also reflected in the performance parameters of the fabricated UV-PD. Figure 7a represents the increasing trend of responsivity from the device with increasing the external applied bias from 0 to − 3 V. The obtained responsivity values are 1.79 A/W and 35.4 A/W at 0 V and − 3 V applied bias respectively by exposing the device with 325 nm UV source at 3 mW optical power. The responsivity value of 35.4 A/W is remarkably high as compared to other bare GaN-based UV-PD as tabulated in Table 1. Though, the recently reported GaN-NT based UV-PD^15^ demonstrated higher responsivity of 88.6 A/W at − 3 V, still, the performance of the device was limited to photo-conductive mode only along with ~ 300 times slower response time. The reduced defect density and built-in electric field in the heterostructure grown in present study might lead towards increased mobility of charge carriers which resulted in faster response along with prominent response in photovoltaic mode. Furthermore, another important parameter i.e., the detectivity of the fabricated device is calculated to be 1.41 × 10^11^ jones at 0 V and 1.83 × 10^10^ jones at − 3 V which divulges that upon applying an external bias, the dark current increases due to thermionic emissions which reduces the detectivity of the developed GaN-NT based device at higher bias condition. Likewise, the QE of the device which is related to sensitivity and defined as the conversion rate of incident photons to generate charge carriers that contribute to current conduction have also been evaluated. Figure 7b demonstrates the impact of variable optical power and applied bias voltage on the QE. In photovoltaic mode, the QE was observed to be increased from 97.41% at 180 µW optical power to 946.75% at 13 mW optical power. Further, QE values have also been evaluated at a fixed optical power of 3 mW and variable applied bias (0 V to − 3 V) condition where QE has been increased from 686.39% (at 0 V) and reached up to 1.35 × 10^4^% (at − 3 V). High value of QE (> 100%) reveals the existence of a photoconductive gain which is greater than unity. In a conventional photodetector the QE is found to be unity i.e., 1 or 100% which means a single photon have generated a charge carrier that have definitely contributed in current conduction. Here, the QE is reported to be very high which could be associated to the following factors: (a) multiple interaction of incident UV photon with the sensing material due to layer by layer stacked hexagonal shaped GaN NTs (offering 3D surface morphology). This unique novel geometry of GaN nanotowers found advantageous to eliminate the reflection losses of incident photons; (b) Efficient collection of generated charge carrier using MSM contact geometry and (c) Reduced defect density of the sensing material (GaN) and reduced trap states^44^. Also, to measure the sensitivity of the fabricated detector, NEP of the device has been determined^45^. The value of NEP was calculated to be 2.42 × 10^–13^ WHz^−1/2^ (at 3 mW, 0 V) which elucidates that the fabricated GaN-NTs based UV-PD can detect a signal of power as low as ~ 200 femto watt with a signal-to-noise ratio of one after the one-half second of averaging.

**Figure 8:**
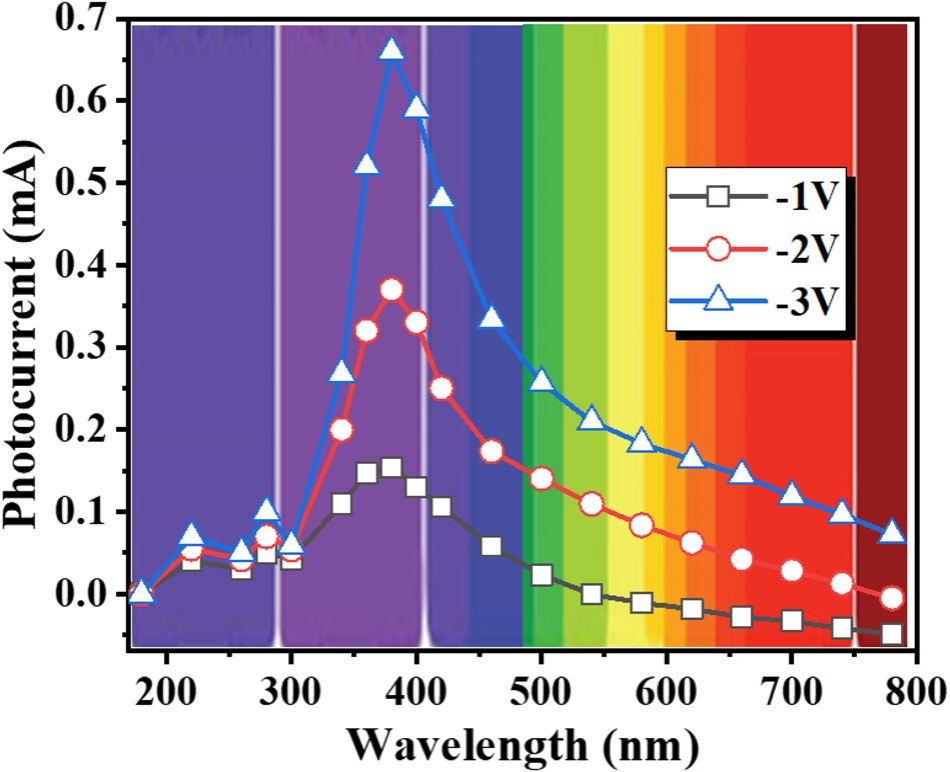
Spectral response characteristic of GaN-NTs based UV-PD from 200 to 800 nm under applied bias.

The spectral selectivity of the fabricated GaN-NTs based UV-PD has also been measured in the range of 200 nm to 800 nm at variable applied bias from − 1 to − 3 V (Fig. 8). Unlike broadband photodetectors^46^, the obtained data demonstrates that the fabricated detector exhibits noteworthy UV light detection selectivity, which designate the detector to find its applications in UV-A spectral range (320–400 nm).

## Conclusion

The fabrication of GaN-NT based UV-PD with independence of operability (in photovoltaic as well as a photoconductive mode) has been demonstrated where the detector yielded enhanced performance due to unique NTs taper-ended geometry. The layered structure of closely packed NTs formed by stacking of uniform hexagonal GaN layers offers the higher active surface area to the incident UV-photons. Thus, the high performance of fabricated detector using GaN NTs has been ascertained from reduce resistance path for charge transport. Thereby, in self-powered mode of operation, the fabricated UV photodetector showcased significantly higher I_Light_/I_Dark_ ratio (> 10^4^), lower dark current (~ 12 nA), remarkable enhancement in photocurrent (~ 7700 fold) as a function of incident optical power, highest responsivity (2.47 A/W), higher detectivity (1.95 × 10^11^ jones), quick response time (~ 433 μs), very high QE (~ 947%) and detection capability of a signal having power as low as 200 femtowatt. Subsequently, the performance of the fabricated UV-PD has also been analysed at photoconductive mode of operation where significantly higher responsivity ~ 35.4 A/W at an applied bias of − 3 V is achieved. The fabricated device with efficient operation and fast transit of charge carriers can effectively be utilized for futuristic optoelectronic devices.

## Supplementary Information

Supplementary Information.
