# Supplementary material for: Fabrication of GaN nano-towers based self-powered UV photodetector
Source: Sci Rep. 2021 May 25;11:10859. doi: 10.1038/s41598-021-90450-w (PMC8149650; doi:10.1038/s41598-021-90450-w)
Supplement: Supplementary file 1 — Supplementary Information. [file 41598_2021_90450_MOESM1_ESM.doc]

**Fabrication of GaN Nano-Towers based Self-Powered UV Photodetector**

**Lalit Goswami^1,2^, Neha Aggarwal^1^, Pargam Vashishtha^1,†^, Shubhendra Jain^1,3,†^, Shruti Nirantar^3^, Jahangeer Ahmed^4^, M. A. Majeed Khan^5^, Rajeshwari Pandey^2^ and Govind Gupta^1,†,*^**

*^1^CSIR-National Physical Laboratory, Dr K.S. Krishnan Road, New Delhi- 110012, India.*

*^2^Department of Electronics & Communication Engineering, Delhi Technological University, New Delhi-110042.*

*^3^Functional Materials and Microsystems Research Group and the Micro Nano Research Facility, RMIT University, Melbourne, VIC 3000, Australia.*

*^4^ Department of Chemistry, College of Science, King Saud University, Riyadh 11451, Saudi Arabia*

*^5^King Abdullah Institute for Nanotechnology, King Saud University, Riyadh, 11451, Saudi Arabia*

***^†^****Academy of Scientific & Innovative Research, CSIR-HRDC Campus, Ghaziabad, Uttar Pradesh - 201002, India.*

^*^Corresponding Author: govind.npl@nic.in; Telephone: +91-1145609503

***Supporting Information:***

The RHEED patterns acquired at various stages during the growth of GaN-NTs/AlN/Si (111) heterostructure are shown in figure S1. Figure S1 (a) shows the RHEED pattern for bare Si (111) substrate which was converted into a strong (7 × 7) reconstructed pattern (shown in figure S1 (b)) by flashing the substrate at a higher temperature. This reveals the formation of atomically clean Si (111) surface to be used for growing the desired heterostructure. The transformation to sharp streaks in RHEED pattern is clearly witnessed during the growth of AlN buffer layer (figure S1 (c)). The spots along with streaks shown in figure S1 (d) for the GaN RHEED pattern, unveils the combination of three – dimensional (3D) and 2D growth of GaN-NTs.


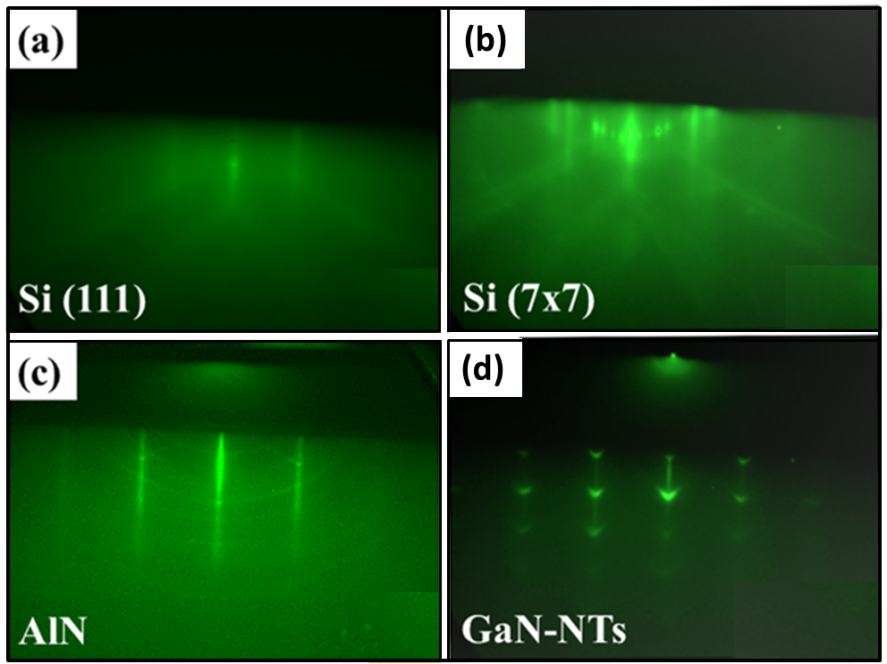


*Figure S1: RHEED Patterns of (a) Si (111) substrate, (b) Si (111) 7 × 7 reconstruction after thermal annealing, (c)AlN buffer layer, (d) GaN-NTs*


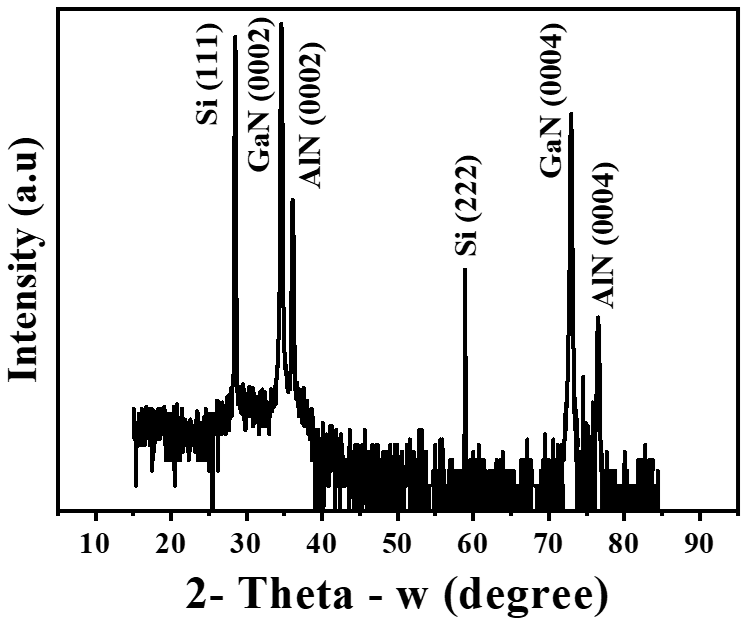


*Figure S2: HRXRD pattern of as grown GaN-NCs/AlN/Si(111) structure.*


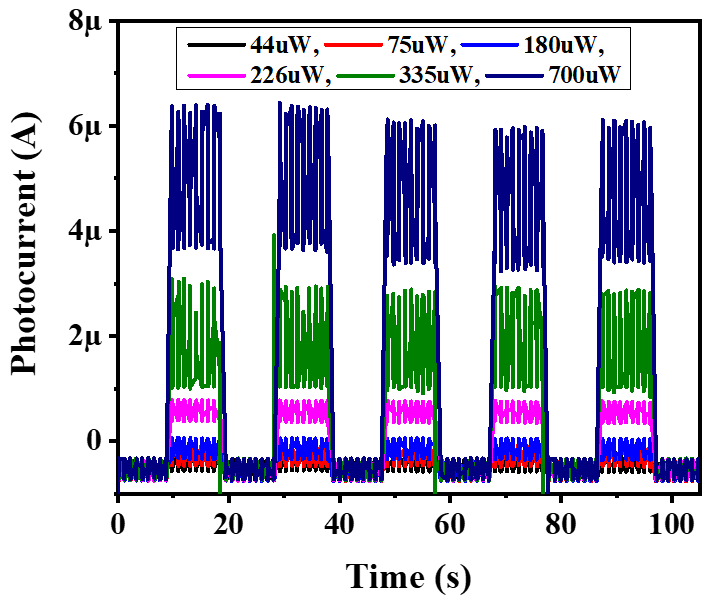


*Figure S3:Time correlated transient photoresponse of fabricated GaN-NCs based UV-PD with applied lower optical power and fixed 325nm UV-laser at 0V.*


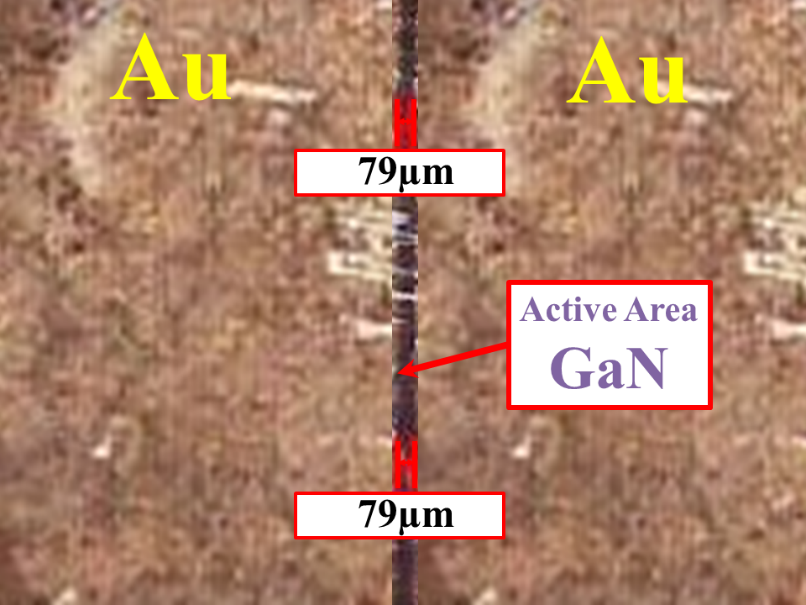


*Figure S4: The optical micrograph of actual UV detection device fabricated in the presented study.*
